# Diagnostic performance of cardiovascular magnetic resonance parametric mapping as per modified Lake Louise Criteria in acute myocarditis: an updated systematic review and meta-analysis

**DOI:** 10.1186/s44348-025-00048-3

**Published:** 2025-06-03

**Authors:** Latika Giri, Roshan Singh, Ahmed Marey, Yupeng Li, Bharath Ambale Venkatesh, Jawdat Abdulla, Stefan Zimmerman, Muhammad Umair

**Affiliations:** 1 Department of Radiology, Kathmandu University School of Medical Sciences, Dhulikhel, Nepal; 2 Department of Radiology, Faculty of Medicine, Alexandria University, Alexandria, Egypt; 3 Department of Political Science and Economics, Rowan University, Glassboro, NJ USA; 4 4Department of Radiology and Radiological Sciences, Johns Hopkins Hospital, Baltimore, MD USA; 5 Department of Cardiology, Amager and Hvidovre Hospital, Hvidovre, Denmark; 6 Deapartment of Radiology, Columbia University Irving Medical Center, NY New York, United States of America

**Keywords:** Myocarditis, Magnetic Resonance Imaging, Extracellular Space, Sensitivity and Specificity, Diagnostic Techniques, Cardiovascular

## Abstract

**Background:**

Cardiovascular magnetic resonance mapping parameters—native T1 mapping, T2 mapping, and extracellular volume (ECV)—are key for diagnosing acute myocarditis under the modified 2018 Lake Louise Criteria (mLLC). This systematic review and meta-analysis evaluated their diagnostic performance and established optimal thresholds for acute myocarditis.

**Methods:**

We reviewed articles published in the past decade utilizing parametric mapping for myocarditis diagnosis. Data on sensitivity, specificity, and area under the curve (AUC) were extracted. Quality assessment was conducted using the QUADAS-2 tool by two independent reviewers.

**Results:**

Eleven studies with 677 patients were included. Native T1 mapping showed sensitivity of 83%, specificity of 86%, diagnostic odds ratio (DOR) of 39, and an AUC of 0.91. T2 mapping had sensitivity of 81%, specificity of 86%, DOR of 25, and an AUC of 0.89. ECV demonstrated sensitivity of 71%, specificity of 81%, DOR of 13, and an AUC of 0.83. Mean control values were 1,039 ± 39.23 ms for native T1 mapping, 57 ± 5.18 ms for T2 mapping, and 31% ± 5.60% for ECV. Optimal thresholds were 1,021 ms for native T1 mapping, 52 ms for T2 mapping, and 28% for ECV based on receiver operating characteristic curves analysis based on 1.5-T scanner value. Native T1 mapping showed the highest diagnostic accuracy. Subgroup analysis found no significant sensitivity differences based on biopsy or clinical criteria.

**Conclusions:**

Parametric mapping, particularly native T1, demonstrated strong diagnostic performance for acute myocarditis compared to T2 mapping and ECV within the modified 2018 Lake Louise Criteria framework. Incorporating these cardiovascular magnetic resonance parameters may improve diagnostic accuracy. Further research is recommended to refine these findings and optimize diagnostic strategies.

**Supplementary Information:**

The online version contains supplementary material available at 10.1186/s44348-025-00048-3.

## Background

Myocarditis, an inflammatory condition of the myocardium, can arise from viral infections, autoimmune disorders, and certain medications [1]. Left untreated, it can lead to severe complications including heart failure, arrhythmias, cardiogenic shock, and dilated cardiomyopathy [2]. Diagnosing myocarditis is challenging due to its heterogeneous and atypical presentation [3]. Endomyocardial biopsy (EMB), while occasionally used, has drawbacks such as invasiveness, sampling limitations, and difficulty in capturing patchy inflammation [4]. Cardiovascular magnetic resonance (CMR) is a common noninvasive modality for detecting myocarditis, employing techniques like early gadolinium enhancement (EGE), T2-weighted images, and late gadolinium enhancement (LGE) from the classical Lake Louise Criteria (LLC) to visualize myocardial inflammation [3, 5, 6]. The addition of quantitative parametric techniques, including native T1 mapping, T2 mapping, and extracellular volume (ECV) in the modified 2018 LLC (mLLC), has further enhanced the diagnostic capability [7–9]. The advantages of parametric mapping include quantitative and objective myocarditis assessment and visualization of global myocardial changes [10, 11]. Several studies have proposed parametric CMR mapping as a gold standard noninvasive technique for myocarditis diagnosis [11, 12]. The values of parametric CMR mapping are influenced by field strength, vendors, and acquisition techniques, leading to a lack of universally reliable cutoff values for diagnosing myocarditis [9, 13, 14]. Our study aims to determine specific native T1 mapping, T2 mapping, and ECV cutoff values that reliably differentiate acute myocarditis from healthy controls. An accurate cutoff for parametric technique like native T1 mapping can help to differentiate acute myocarditis patients from healthy controls and narrow nondefinitive diagnosis cases [6]. Both native T1 and T2 mapping are sufficient as single markers and can increase diagnostic confidence when used together [15]. Additionally, combining parametric techniques, such as native T1 and T2 mapping along with ECV, has proven beneficial in increasing sensitivity and specificity for myocarditis detection [16]. This study evaluates the diagnostic performance of individual CMR mapping parameters (T1, T2, and ECV) in detecting myocarditis, focusing on their independent diagnostic value within the mLLC framework and without incorporating combined parametric techniques.

## Methods

We conducted a systematic review and meta-analysis to assess the diagnostic performance of parametric CMR parameters—native T1 mapping, T2 mapping, and ECV for myocarditis diagnosis (individual or combined) and provide their respective cutoff values in acute myocarditis cases. The study adhered to PRISMA (Preferred Reporting Items for Systematic Reviews and Meta-Analyses) guidelines, and the protocol was registered in the PROSPERO (International Prospective Register of Systematic Reviews) database (No. CRD42023484275).

### Study search and selection criteria

**Fig. 1:**
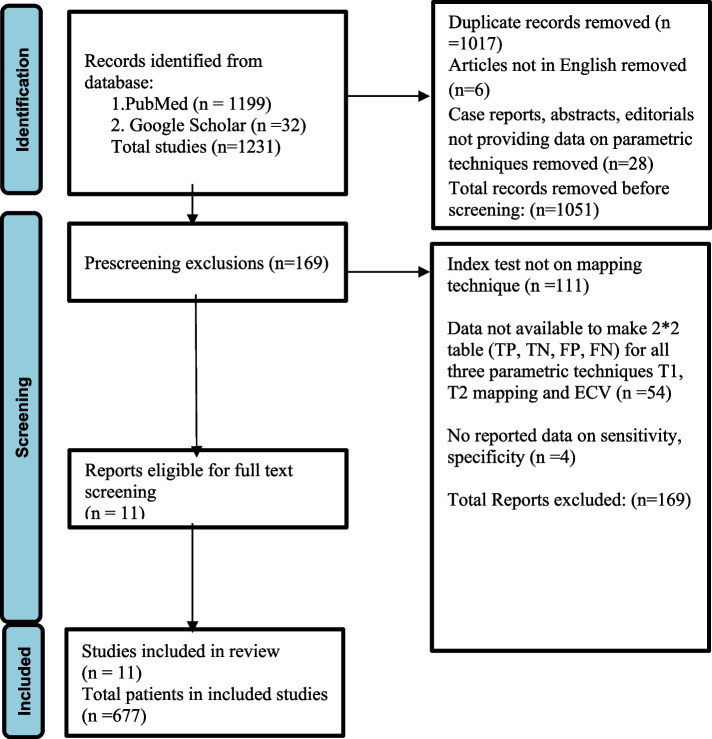
PRISMA (Preferred Reporting Items for Systematic Reviews and Meta-Analyses) flowchart for studies selection

Two reviewers (LG and RS) independently searched PubMed and Google Scholar for relevant studies using predefined keywords:"myocarditis"AND “magnetic resonance imaging” AND “Lake Louise Criteria AND"Modified Lake Louise Criteria,” AND"New Lake Louise Criteria,"AND"2018 Lake Louise Criteria") AND “ECV.” Studies from 2014 to 2024 were included. A comprehensive literature search was conducted in PubMed and Google Scholar from January 1, 2014, up to January 1, 2024. The final selection of studies and data synthesis was completed on March 1, 2024. Inclusion criteria encompassed prospective and retrospective studies evaluating the diagnostic accuracy of the three parametric CMR techniques (native T1 mapping, T2 mapping, and ECV) compared to EMB or accurate clinical assessment as reference. Despite the introduction of the mLLC in 2018, our inclusive approach considered studies dating back to 2014, as many authors had incorporated mapping techniques and ECV in their research even before the formal introduction of the mLLC in 2018. Exclusion criteria eliminated case reports, reviews, and studies lacking sufficient data on calculating sensitivity or specificity. Disagreements were resolved through discussion or involvement of a third reviewer (MU). Risk of bias was assessed using the revised Quality Assessment of Diagnostic Accuracy Studies (QUADAS-2) tool (Fig. 1).

### Statistical analysis

The meta-analysis was performed using R ver. 4.3.2 (R Foundation for Statistical Computing). Pooled diagnostic odds ratios (DORs) and 95% confidence intervals were calculated for single parametric techniques and, if available, for combined parametric mapping. Diagnostic accuracy was evaluated using the area under the curve (AUC) from summary receiver operating characteristic (ROC) curves. A univariate random effects model assessed pooled sensitivity and specificity, and statistical heterogeneity was evaluated using the I^2^ statistic, with significant heterogeneity defined as I^2^ > 60%. ROC analysis was performed to calculate the optimal thresholds of native T1 mapping, T2 mapping, and ECV along with their respective AUC, sensitivities, and specificities. Pooled mean native T1 mapping, T2 mapping, and ECV values were calculated by combining means and standard deviations from multiple studies, and optimal thresholds were calculated by ROC curves. Subgroup analyses were conducted based on study reference standards. Funnel plots and Egger regression test were used to inspect and test for publication bias. Continuous variables were expressed as means ± standard deviations, and categorical values as numbers and percentages.

## Results

### Search results

A total of 1,199 articles were initially identified, with 180 undergoing full-text review. Ultimately, 11 studies from 2014 to 2022 were included in the meta-analysis (Tables 1 and 2) [7, 8, 14–17, 19, 20, 25, 26, 28]. These comprised seven prospective [7, 8, 14–16, 19, 28] and four retrospective designs [17, 20, 25, 26], primarily conducted in Germany.

**Table 1:** Summary of study characteristics and demography of included studies

Study	Study design	No. of patients	Age (yr)^a^	Sex (%)		Baseline presentation	(Reference standard)^d^	CMR timing post-symptom onset (day)^b^
				Male	Female			
Huber et al. [17] (2018)	Retrospective cohort with case–control element	60	45 (29–61)	60	40	Acute^c^	Clinical	≤ 7
Radunski et al. [25] (2014)	Retrospective case–control	125	44	76	24	Acute^c^	Clinical	3
Isaak et al. [26] (2021)	Retrospective case–control	56	17 (14–20)	77	23	Acute^c^	EMB	5
Palmisano et al. [28] (2020)	Prospective case–control	43	39 (28–46)	51	49	Acute^c^	Clinical	< 14
Luetkens et al. [19] (2016)	Prospective case–control	84	44.9 (26.2–63.17)	50	50	Acute^c^	Clinical	7
Li et al. [20] (2021)	Retrospective cohort with case–control element	73	32 (18–50)	71.2	28.8	Acute^c^	EMB	10 ± 4
Lurz et al. [8] (2016)	Prospective cohort with case–control element	129	40	83	17	Acute^c^	EMB	< 14
von Knobelsdorff-Brenkenhoff et al. [15] (2017)	Prospective cohort with case–control element	36	24.5	78	22	Acute^c^	Clinical	< 14
Luetkens et al. [7] (2019)	Prospective cohort with case–control element	66	41 (23–64)	72.5	27.5	Acute^c^	Clinical	9
Dabir et al. [14] (2014)	Prospective cohort with case–control element	80	38 (22–54)	77	23	Acute^c^	Clinical	< 28
Brendel et al. [16] (2022)	Prospective cohort with case–control element	48	48 (30–63)	44	56	Acute^c^	EMB	6

CMR, cardiovascular magnetic resonance; EMB, endomyocardial biopsy

^a^Values are presented as median only or median (interquartile range). ^b^Values are presented as range, median only, or mean ± standard deviation. ^c^Acute myocarditis (patients presenting with symptoms within 28 days). ^d^Clinical criteria is based on the European Society of Cardiology guidelines (symptoms, elevated troponin, and cardiovascular magnetic resonance imaging/EMB confirmation), while the EMB criteria required histologic evidence of inflammation (Dallas criteria) and/or immunohistochemistry (CD3/CD45 lymphocytes)

**Table 2:** CMR characteristics of included studies

Study	Native T1 mapping (msec)		T2 mapping (msec)		ECV (%)		Technique		CMR type
	Control	Case	Control	Case	Control	Case	Native T1 mapping	T2 mapping	
Huber et al. [17] (2018)	965 (940–990)	1,044 (981–1,107)	48 (46–50)	53 (49–57)	22 (19–25)	24 (17–31)	MOLLI	bSSFP	1.5 T (Siemens)
Radunski et al. [25] (2014)	1,051 (1,010–1,063)	1,098 (1,057–1,139)	55 (54–60)	61 (58–65)	25 (24–27)	31 (28–34)	MOLLI	NA	1.5 T (Philips)
Isaak et al. [26] (2021)	962 (945–979)	1,031 (985–1,077)	51 (49–53)	58 (53–63)	26.5 (23.7–29.3)	29.2 (23.3–35.1)	MOLLI	GRASE	1.5 T (Philips)
Palmisano et al. [28] (2020)	1,008 (988–1,033)	1,093 (1,050–1,201)	47 (46–47.6)	55 (52–59)	25 (24–26)	30 (27–32)	MOLLI	NA	1.5 T (Philips)
Luetkens et al. [19] (2016)	966.9 (939.1–994.7)	1,048.6 (996.7–1,100.5)	52.42 (49.86–54.98)	60.43 (52.96–67.9)	27.68 (21.86–33.5)	34.47 (25.95–43.99)	MOLLI/ShMOLLI	GRASE	1.5 T (Philips)
Li et al. [20] (2021)	1,195 (1,152–1,238)	1,252 (1,210–1,294)	54.5 (50.8–58.2)	63.2 (57.1–69.3)	29.3 (25.2–33.4)	32.7 (29.4–36)	MOLLI	GRASE	3 T (Philips)
Lurz et al. [8] (2016)	1,044 (1,002–1,086)	1,113 (1,046–1,180)	56.9 (49.7–64.1)	62.2 (57.7–66.7)	31.8 (26.9–36.7)	37.2 (30.7–43.7)	MOLLI	NA	1.5 T (Siemens)
von Knobelsdorff-Brenkenhoff et al. [15] (2017)	975 (957–1,004)	1,004 (988–1,048)	50.2 (49.2–52)	55.1 (53.3–57.2)	24 (24–25)	26 (25–28)	MOLLI	bSSFP	1.5 T (Philips)
Luetkens et al. [7] (2019)	965.8 (940.7–990.9)	1,047.0 (993.2–1,100.8)	52.8 (50.4–55.2)	61.8 (53.6–70)	26.1 (21.9–30.3)	28.6 (23.3–33.9)	MOLLI	GRASE	1.5 T (Philips)
Dabir et al. [14] (2014)	958.9 (936.4–981.4)	1,027.2 (977.9–1,076.5)	51.6 (49.7–53.5)	58 (52–64)	27.7 (24.5–30.9)	32 (25.6–38.4)	MOLLI	GRASE	1.5 T (Philips)
Brendel et al. [16] (2022)	1,015 (1,015–1,015)	1,069 (1,024–1,127)	50 (50–50)	53 (52–56)	30 (30–30)	33 (31–35)	MOLLI	bSSFP	1.5 T (Philips)

Values are presented as median (interquartile range). The optimal diagnostic thresholds (determined using ROCs curve for 1.5-T scanner value only) were 1,021 ms for native T1 mapping, 52 ms for T2 mapping, and 28% for ECV. The ROC AUC was 0.88 for native T1 mapping, 0.94 for T2 mapping, and 0.79 for ECV. The sensitivity values were 91% for native T1 mapping, 92% for T2 mapping, and 82% for ECV. The specificity values were 80% for native T1 mapping, 82% for T2 mapping, and 83% for ECV

CMR, cardiovascular magnetic resonance; ECV, extracellular volume; MOLLI, modified Look-Locker inversion recovery; bSSFP, balanced steady-state free precession; NA, not available; GRASE, gradient- and spin-echo; ShMOLLI, shortened modified Look-Locker inversion recovery; ROC, receiver operating characteristic; AUC, area under the curve

### Diagnostic accuracy results

**Fig. 2:**
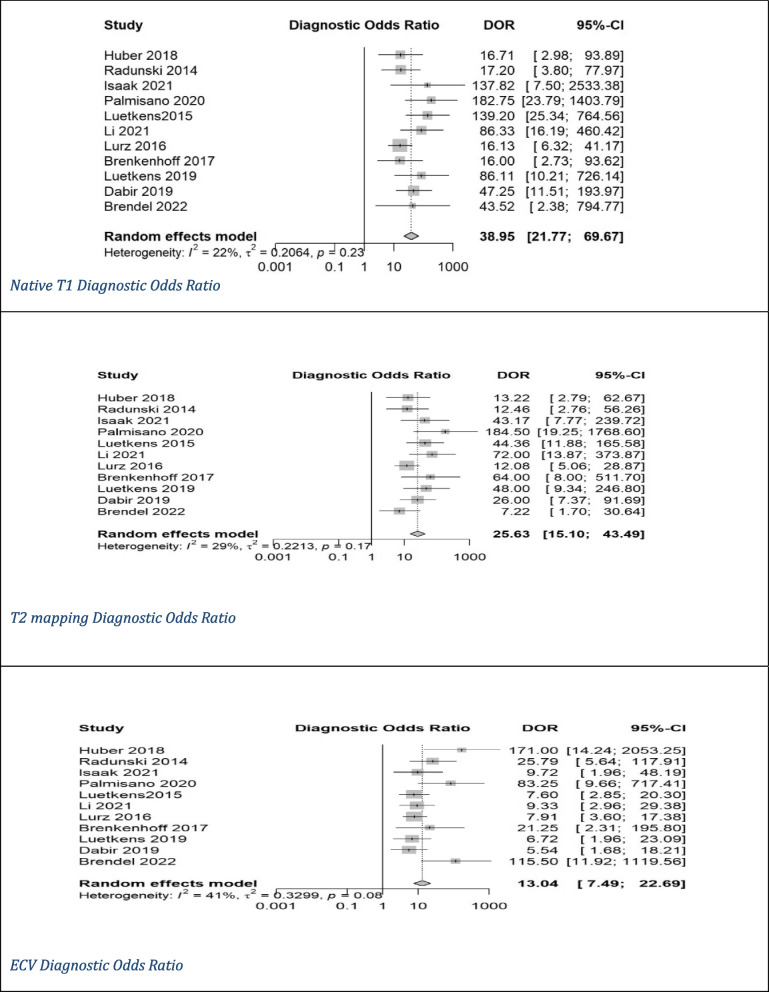
Forest plot of diagnostic odds ratio (DOR) of (**A**) native T1 mapping, (**B**) T2 mapping, and extracellular volume for myocarditis diagnosis. CI, confidence interval

**Fig. 3:**
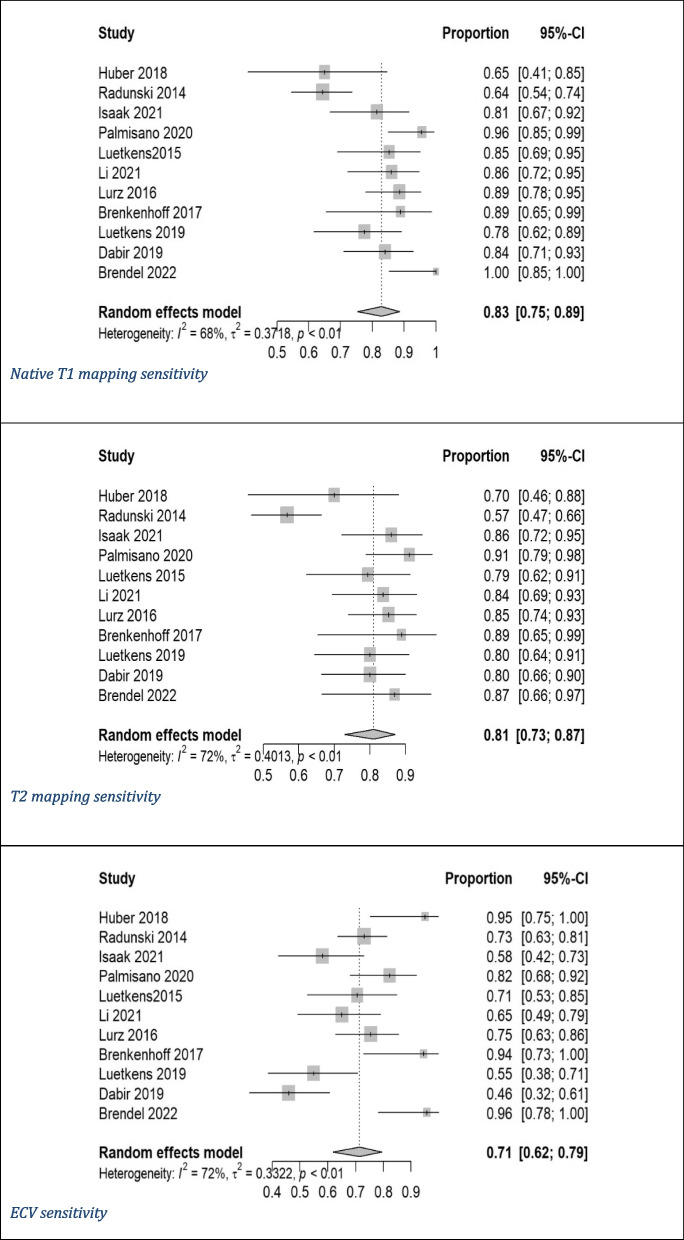
Sensitivity forest plot of (**A**) native T1 mapping, (**B**) T2 mapping, and (**C**) extracellular volume for diagnosis of myocarditis. CI, confidence interval

**Fig. 4:**
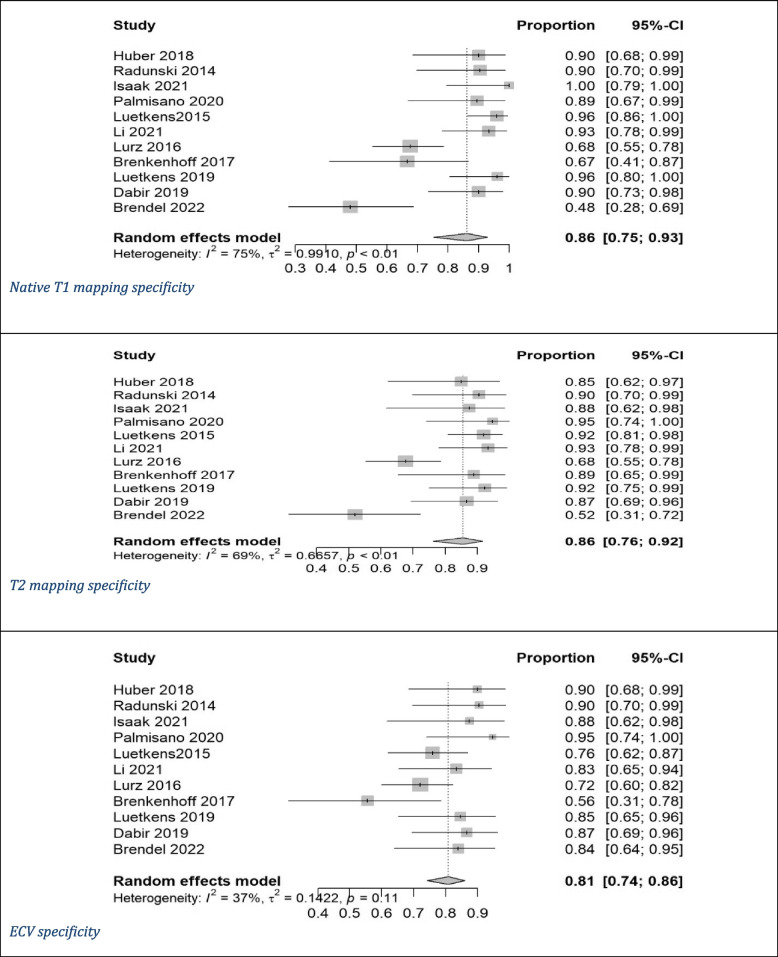
Specificity forest plot of (**A**) native T1 mapping, (**B**) T2 mapping, and (**C**) extracellular volume for diagnosis of myocarditis

**Fig. 5:**
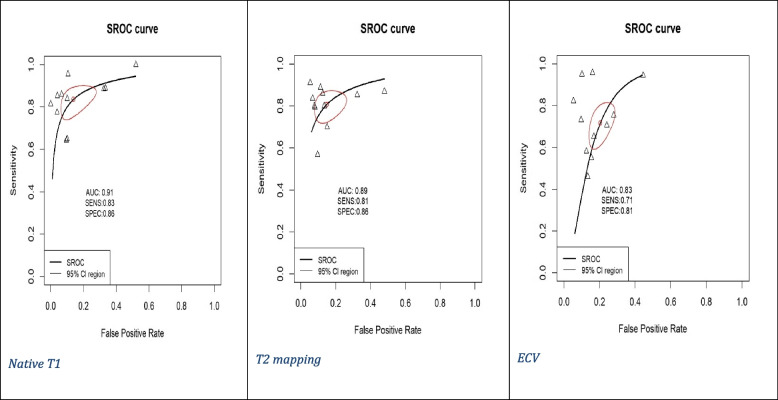
Plot of symmetric summary receiver operating curve characteristic (SROC) of (**A**) native T1 mapping, (**B**) T2 mapping, and (**C**) extracellular volume for diagnosis of myocarditis. AUC, area under the curve; SENS, sensitivity; SPEC, specificity; CI, confidence interval

Pooled mean T1 time was 1,021 ms, and pooled mean T2 time was 52 ms for myocarditis cases based on values of 1.5-T scanner. ECV cutoffs ranged from 22% to 34.47%, and postcontrast T1 timing ranged from 10 to 15 min. Out of the 11 studies, only 2 studies used CMR from Siemens [8, 17]. Ten studies utilized modified Look-Locker inversion recovery (MOLLI) for native T1 mapping [7, 8, 14–17, 20, 25, 26], with one using both MOLLI and shortened MOLLI (ShMOLLI) [19]. We had five studies utilizing gradient- and spin-echo (GRASE) [7, 14, 16, 19, 20], three using balanced steady-state free precession (bSSFP) as a T2 mapping technique [15–17], and three studies did not report the T2 mapping technique utilized [8, 25, 28]. Diagnostic accuracy for native T1 mapping, T2 mapping, and ECV was reported in all studies (Table 1) [7, 8, 14–17, 19, 20, 25, 26, 28]. Native T1 mapping demonstrated the highest sensitivity (83%) and specificity (86%) among the evaluated parameters, with significant heterogeneity (I^2^) of 69% for sensitivity and 75% for specificity. It also had a DOR of 39 and an AUC of 0.91. T2 mapping showed similar sensitivity (81%) and specificity (86%) with I^2^ of 72% and 69%, respectively, and a DOR of 25 with an AUC of 0.89. ECV exhibited moderate sensitivity (71%) and specificity (81%) with I^2^ of 72% and 37%, respectively, along with a lower DOR of 13 and an AUC of 0.83 (Figs. 2, 3, 4 and 5) [7, 8, 14–17, 19, 20, 25, 26, 28].

Although only five studies reported diagnostic performance for combined parametric techniques, precluding a meta-analysis, we present these combinations and their respective sensitivity, specificity, and AUC values in Table 3 to reflect their clinical relevance [16, 17, 20, 25, 26] (Tables 4 and 5).

**Table 3:** Diagnostic criteria for included studies

Study	Reference standard^a^
Huber et al. [17] (2018)	Clinical
Radunski et al. [25] (2014)	Clinical
Isaak et al. [26] (2021)	EMB
Palmisano et al. [**] (2020)	Clinical
Luetkens et al. [19] (2016)	Clinical
Li et al. [20] (2021)	EMB
Lurz et al. [8] (2016)	EMB
von Knobelsdorff-Brenkenhoff et al. [15] (2017)	Clinical
Luetkens et al. [7] (2019)	Clinical
Dabir et al. [14] (2014)	Clinical
Brendel et al. [16] (2022)	EMB

EMB, endomyocardial biopsy

**Table 4:** Diagnostic accuracies and heterogeneity (I^2^) of native T1 mapping, T2 mapping, and ECV

Parameter	Sensitivity (%)	I^2^ (%)	Specificity (%)	I^2^ (%)	DOR	AUC
Native T1 mapping	83	69	86	75	39	0.91
T2 mapping	81	72	86	69	25	0.89
ECV	71	72	81	37	13	0.83

ECV, extracellular volume; DOR, diagnostic odds ratio; AUC, area under the curve

**Table 5:** Diagnostic performance of combined CMR parametric mapping techniques (native T1, T2, ECV) in acute myocarditis across five included studies

Study	Combined CMR technique	Sensitivity (%)	Specificity (%)	AUC
Li et al. [20] (2021)	Native T1 mapping + T2 mapping	92	89	0.96
Huber et al. [17] (2018)	Native T1 mapping + ECV	88	91	0.94
Radunski et al. [25] (2014)	Native T1 mapping + T2 mapping	85	93	0.95
Isaak et al. [26] (2021)	Native T1 mapping + T2 mapping + ECV	94	88	0.97
Brendel et al. [16] (2022)	Native T1 mapping + T2 mapping	89	90	0.93

CMR, cardiovascular magnetic resonance; ECV, extracellular volume; AUC, area under the curve

**Fig. 6:**
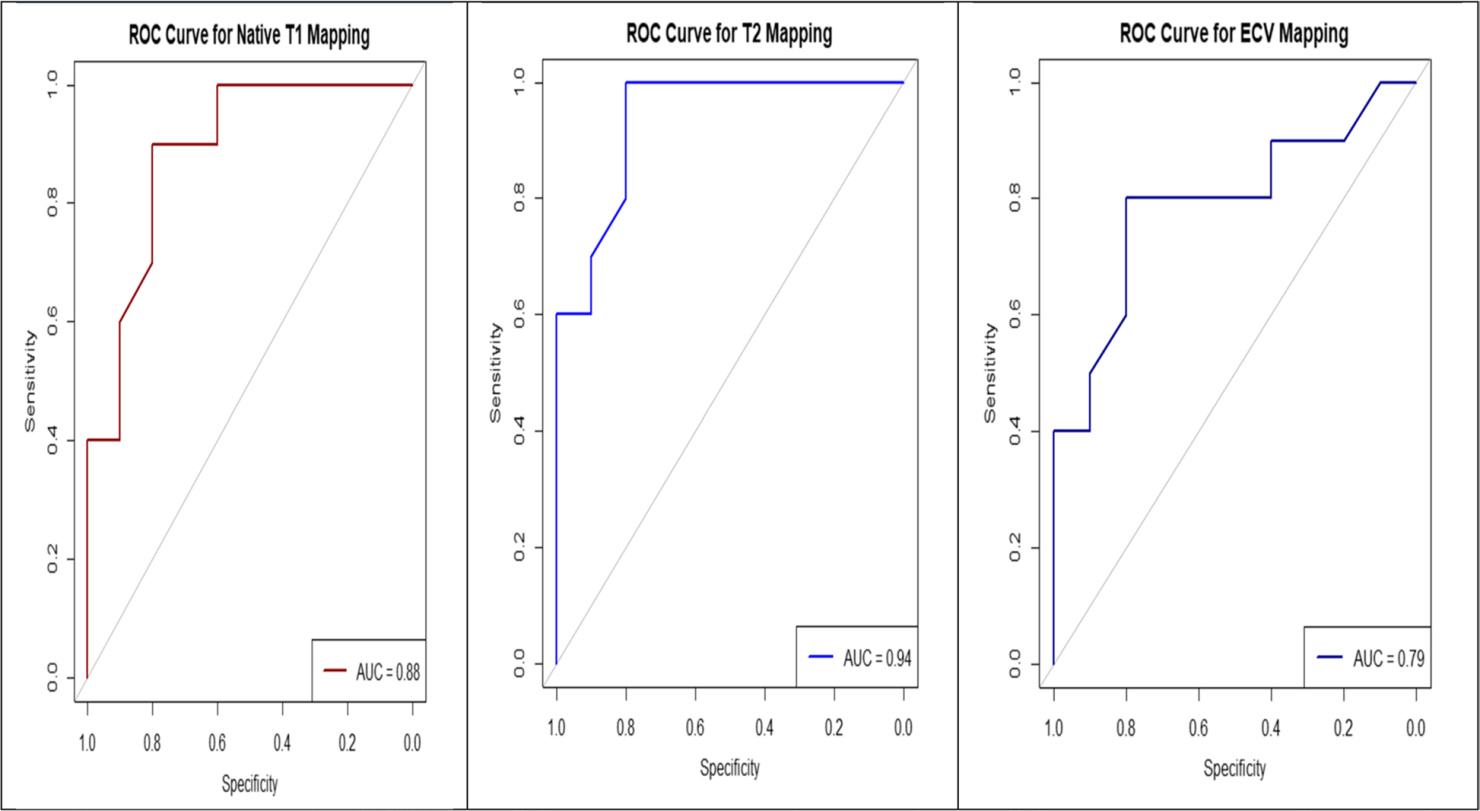
Receiver operating characteristic curve for (**A**) native T1 mapping, (**B**) T2 mapping, and (C) extracellular volume threshold in diagnosing myocarditis. AUC, area under the curve; SENS, sensitivity

The optimal cutoff for native T1 mapping was 1,021 ms (ROC AUC, 0.88; sensitivity, 91%; specificity, 80%). For ECV, the threshold was 28% (ROC AUC, 0.79; sensitivity, 82%; specificity, 83%) and T2 mapping had an optimal threshold of 52 ms (ROC AUC, 0.94; sensitivity, 92%; specificity, 82%) based on values obtained on the 1.5-T scanner (Fig. 6).

Subgroup analyses revealed no significant differences in diagnostic accuracy based on clinical criteria or EMB reference standards. Meta-regression showed no significant sensitivity differences for native T1, T2, and ECV (*P* > 0.05), though native T1 mapping specificity varied significantly (*P* < 0.05) (Table 6) (Supplementary Table 1) [8, 28]. The QUADAS-2 risk of bias assessment is provided in Table 7 [7, 8, 14–17, 19, 20, 25, 26]. Funnel plot examination and Egger test indicated no significant publication bias for T1, T2, and ECV parameters (all *P* > 0.05). Egger regression test revealed no significant publication bias for native T1 mapping (*P* = 0.15), T2 mapping (*P* = 0.22), or ECV (*P* = 0.30).

**Table 6:** Results of meta-regression and subgroup analyses based on reference standard of studies

Reference standard	Sensitivity (%)	*P*-value	Specificity (%)	*P*-value
Native T1 mapping		0.770		0.001
Clinical (Lurz et al. [8])	79 (70–87)		92 (87–96)	
EMB (Palmisano et al. [28])	88 (82–93)		70 (50–84)	
T2 mapping		0.205		0.076
Clinical (Lurz et al. [8])	78 (67–87)		90 (84–93)	
EMB (Palmisano et al. [28])	85 (79–90)		77 (56–90)	
ECV		0.174		0.102
Clinical (Lurz et al. [8])	67 (55–78)		84 (78–89)	
EMB (Palmisano et al. [28])	81 (64–91)		74 (62–84)

**Table 7:** QUADAS-2 risk of bias assessment

Study	**Risk of bias assessment**			
	Patient selection	Index test	Reference standard	Flow and timing
Huber et al. [17] (2018)	Unclear	Unclear	Low	Low
Radunski et al. [25] (2014)	Unclear	Low	Low	Low
Isaak et al. [26] (2021)	Low	Unclear	Low	Low
Palmisano et al. [28] (2020)	Low	Low	Low	Low
Luetkens et al. [19] (2016)	Low	Unclear	Low	Low
Li et al. [20] (2021)	Low	Unclear	Low	Low
Lurz et al. [8] (2016)	Low	Unclear	Low	Low
von Knobelsdorff-Brenkenhoff et al. [15] (2017)	Low	Low	Low	Low
Luetkens et al. [7] (2019)	Low	Low	Low	Low
Dabir et al. [14] (2014)	Low	Low	Low	Low
Brendel et al. [16] (2022)	Low	Unclear	Low	Low

QUADAS-2, revised Quality Assessment of Diagnostic Accuracy Studies

## Discussion

Our study focused on evaluating the diagnostic accuracies of individual parametric mapping techniques for acute myocarditis. Prolonged T1 values, attributed to cellular edema, increased extracellular space, inflammation, and myocyte necrosis, are consistently observed in myocarditis cases [18, 19]. Native T1 mapping showed the highest accuracy among the studied techniques, consistently demonstrating good diagnostic performance (AUC, 0.90; DOR, 39) compared to T2 mapping (AUC, 0.88; DOR, 25) and ECV (AUC, 0.83; DOR, 13). This aligns with findings by several other studies indicating superior diagnostic efficacy of T1 mapping techniques over original LLC 2009 techniques (T2-weighted imaging, LGE, and EGE) [15, 18–20].

It is well known that native T1 values can be affected by mapping sequence used and field strength [21]. In the current study, however, we found relative uniformity among the studies included in the meta-analysis with respect to these parameters. Out of 11 studies, 10 used the MOLLI sequence, and 10 studies used a 1.5-T strength magnet. Hence the interstudy variation in native T1 values in the current study are likely secondary to other factors. Caution should be taken in applying the current results if not using MOLLI on a 1.5-T scanner. For native T1 mapping an optimal cutoff of 1,021 ms (ROC AUC, 0.82; sensitivity, 91%; specificity, 73%) was found. However, this is a statistical threshold, and its clinical significance still needs to be completely determined.

ECV, derived from precontrast and postcontrast T1 and hematocrit values, exhibited promise with an AUC of 0.83. However, it is well known that edema itself elevates ECV, which may make the utility of ECV even less reliable among the hyper acute and acute phase of inflammation [21]. A median time of less than 14 days from symptom onset to CMR acquisition in our analysis likely contributed to ECV's lower sensitivity, as observed in a study by Luetkens et al. [7]. ECV values vary with CMR sequences but remain unaffected by field strength, distinguishing them from native T1 mapping and T2 mapping [22]. Li et al. [20] reported an ECV of 32.7% (range, 29.4%–36.0%) for acute myocarditis cases, and 29.3% (range, 25.2%–33.4%) for controls (*P* < 0.01) using a 3-T magnet. In comparison, Dabir et al. [14] found an ECV of 32.0% (range, 25.6%–38.4%) for myocarditis patients and 27.7% (range, 24.5%–30.9%) for controls (*P* = 0.01) using a 1.5-T magnet. ECV had an optimal threshold of 28% (ROC AUC, 0.79; sensitivity, 82%; specificity, 73%) however it is a statistical measure.

Myocardial T2 relaxation time emerged as a promising marker, with significantly elevated values in patients during acute presentation compared to controls. Myocardial T2 relaxation time, closely linked to free tissue water content, with significantly elevated values in acute myocarditis patients [20]. In our study, T2 mapping demonstrated comparable diagnostic efficacy to native T1 mapping, with an AUC of 0.88. Variations in T2 mapping techniques and thresholds necessitate consideration of acquisition types, patient populations, and clinical contexts [23, 24]. A statistical analysis identified an optimal threshold of 54 ms for detecting myocarditis using T2 mapping, with a ROC AUC of 0.93, 82% sensitivity, and 82% specificity.

This meta-analysis includes combined mapping techniques from recent studies. This combined approach is most reflective of clinical practice. Five studies reported enhanced diagnostic outcomes by use of several different integrated mapping techniques, such as native T1 and T2 or native T1 and ECV [16, 17, 20, 25, 26]. A study by Li et al. [20] demonstrated high diagnostic performance when combining native T1 and T2 mapping over a single parameter of mLLC. A small number of studies precluded a pooled analysis in our meta-analysis. Subgroup analysis with fewer than four studies would be unreliable due to low statistical power to detect true differences between subgroups. The study supports the notion that native T1 and T2 mapping allow for a reliable distinction between injured and normal myocardium but may struggle to discriminate between acute myocarditis and other noninflammatory cardiomyopathy or myocarditis with a chronic presentation.

The diagnostic accuracy of parametric mapping techniques has been established to be superior to older techniques. Available parametric mapping evaluations demonstrate that at least one of the main mLLC criteria can effectively diagnose myocarditis with little less accuracy as an alternative to EMB (88% sensitivity for EMB > 80% for parametric mapping in CMR) [27].

### Limitations

The results of this meta-analysis should be interpreted cautiously due to possible selection and reference biases. Although most studies used 1.5-T scanners, inclusion of even a single 3-T study [20] might introduce variability due to known differences in baseline mapping values. This may limit the generalizability of our results and should be considered when applying threshold values in clinical practice. We evaluated only the diagnostic performance of individual parametric techniques due to inadequate studies on combined parametric approach. However individual parameters are rarely used in isolation in clinical practice. Most studies included in our analysis exhibited variability in population, study design, patient characteristics, observer interpretations, software variation, and CMR acquisition protocols. EMB was utilized as reference in four studies, while the other studies relied on clinical diagnoses and case–control designs. Some studies did not report local normal data for native T1 mapping, potentially affecting diagnostic accuracy. Variations in T2 mapping techniques (GRASE or SSFP) and CMR vendors (Philips or Siemens) at 1.5-T field strength may limit generalizability. While pooled thresholds for T1/T2 mapping (e.g., T1 > 1,021 ms) provide a diagnostic framework, their clinical application requires local validation due to vendor-, field strength-, and sequence-dependent variability. In contrast, ECV thresholds (e.g., 28%) demonstrate greater reproducibility and should be prioritized in standardized protocols.

The small number of studies on updated LLC combined mapping parameters precluded a reliable sensitivity analysis, particularly for combined T1 and T2 mapping techniques. Our meta-analysis evaluated parametric mapping (T1, T2, ECV) per mLLC. However, excluding T2-weighted imaging and LGE limits direct applicability to clinical practice, where these tools are routinely combined. Future studies should assess integrated protocols incorporating both parametric mapping and conventional techniques.

### Clinical implications and future directions

Our investigation underscores native T1 mapping as the most effective modality for diagnosing acute myocarditis, with T2 mapping and ECV presenting viable alternatives. Future studies should refine diagnostic protocols based on patient presentation timing, standardize CMR methods across large, matched cohorts, and consider diagnostic network meta-analyses to optimize approaches. This approach aims to reduce diagnostic costs and clinical workload associated with myocarditis, facilitating more precise and efficient diagnosis.

## Conclusions

The utilization of CMR based parametric mapping emerges as a valuable diagnostic strategy for acute myocarditis with native T1 mapping exhibiting highest diagnostic accuracy across the board, followed by T2 mapping and ECV. The pooled optimal threshold values of each native T1 mapping, T2 mapping, and ECV obtained from ROC aid in adequate diagnosis of myocarditis. We recommend further studies to explore the diagnostic performance of combined parametric mapping techniques.

## Supplementary Information

Supplementary Materials 1. Table S1. Subgroup analysis by study design (prospective vs. retrospective).

## Data Availability

No datasets were generated or analysed during the current study.

## Abbreviations

AUC: Area under the curve
bSSFP: Balanced steady-state free precession
CI: Confidence interval
CMR: Cardiovascular magnetic resonance
DOR: Diagnostic odds ratio
ECV: Extracellular volume
EGE: Early gadolinium enhancement
EMB: Endomyocardial biopsy
GRASE: Gradient- and spin-echo
LGE: Late gadolinium enhancement
LLC: Lake Louise Criteria
MOLLI: Modified Look-Locker inversion recovery
PRISMA: Preferred Reporting Items for Systematic Reviews and Meta-Analyses
PROSPERO: International Prospective Register of Systematic Reviews
ROC: Receiver operating characteristic
ShMOLLI: Shortened modified Look-Locker inversion recovery
SSFP: Steady-state free precession
