# Supplementary material for: Diagnostic performance of cardiovascular magnetic resonance parametric mapping as per modified Lake Louise Criteria in acute myocarditis: an updated systematic review and meta-analysis
Source: J Cardiovasc Imaging. 2025 Jun 3;33:5. doi: 10.1186/s44348-025-00048-3 (PMC12131415; doi:10.1186/s44348-025-00048-3)
Supplement: Supplementary file 1 — Supplementary Materials 1. Table S1. Subgroup analysis by study design (prospective vs. retrospective). [file 44348_2025_48_MOESM1_ESM.docx]

Supplementary Table 1: Subgroup Analysis by Study Design (Prospective vs. Retrospective)

| Parameter | Subgroup | Sensitivity (95% CI) | Specificity (95% CI) | I² (Sensitivity) | I² (Specificity) | p-value (Subgroup Differences) |
| --- | --- | --- | --- | --- | --- | --- |
| Native T1 | Prospective | 81% (70–87%) | 85% (80–90%) | 68% | 70% | Sensitivity: *p* = 0.23 |
|  | Retrospective | 86% (82–93%) | 87% (83–91%) | 72% | 80% | Specificity: *p* = 0.12 |
| T2 Mapping | Prospective | 78% (67–87%) | 84% (77–89%) | 70% | 65% | Sensitivity: *p* = 0.18 |
|  | Retrospective | 85% (79–90%) | 88% (83–92%) | 75% | 73% | Specificity: *p* = 0.09 |
| ECV | Prospective | 68% (55–78%) | 79% (72–85%) | 70% | 35% | Sensitivity: *p* = 0.15 |
|  | Retrospective | 75% (64–83%) | 83% (75–89%) | 74% | 40% |  |

Retrospective studies showed marginally higher sensitivity/specificity than prospective studies, but differences were not statistically significant (*p* > 0.05).

High heterogeneity (I² > 60%) persisted across all parameters.

Supplementary Table 2: Subgroup analyses by field strength(only 1 study on 3T), vendor(only 2 studies on a different vendor) not done because of very less studies (<4) in each subgroup each.
